# College of Physicians (First Meeting)

**Published:** 1830-04-01

**Authors:** 


XXXVIII.

College of Physicians.

The evening meetings of this splendid edifice opened with unusual éclat on Monday night, the 8th of February. The Prime Minister and the Lord Chancellor sat on the left and right of the President, and a constellation of rank and talent crowded the spacious library, and even the galleries. Sir Henry Halford read, or rather spoke, a paper with his usual eloquence, and from his well known store of classical and medical erudition. It was admirably adapted for the mixed audience to which it was addressed, being chiefly on the prophetic power attributed by many philosophers and poets of antiquity to the last moments of existence. That many people approach the very goal of human life—the portals of another state of being, or, if the materialists will have it so, the verge of annihilation, with a perfect integrity of their intellectual functions, is well known to medical practitioners. This happens more particularly when the brain is not the principal seat of the disease. For our own parts, we have never seen a single instance where the functions of that important organ resumed any thing like tranquillity or clearness, when the disorganization of its material structure was the cause of death. The varied and extensive practice, however, of Sir H. Halford has afforded him the opportunity of observing this rare phenomenon. The following is an outline of the case which was offered by the talented President as an illustration.

"A young gentleman who had been using mercury caught cold while under its influence, and became affected with fever. On the seventh day, when Sir Henry was first called in, he was in a state of the highest excitement—threatening those around him, and not to be approached without increasing his irritation to fury. He was put under restraint, and tartarized antimony administered at intervals, in doses of a grain each time. On the eleventh day from the commencement of the attack, he had become quite calm, and to those about him he seemed to be much better. It was observed that he had repeatedly said he should die, and had talked with the utmost composure of his affairs, giving directions for their arrangement. He sent messages to his absent friends, and spoke of a sister recently dead, as one whom he was about immediately to follow. In answer to his interrogations, Sir Henry found that he had not slept anterior to this quietude, and that his pulse was quicker than ever. He then became satisfied that the improvement was but in appearance—that it was "a lightning before death"—and that the hours of his patient were numbered. He died that night."*

As no dissection is given of this case, it is by no means certain that the cause of death was in the brain, though its function was so dreadfully disturbed during the course of the disease. The description of the Kausos, by Aretæus, was commented on by the President in elegant and eloquent terms, and it is hardly necessary to state, that the prophetic power attributed to the patient by the ancient Greek physician was not credited by the moderns. Numerous and very interesting quotations were made from poets, in later as well as earlier periods where the belief of dying inspiration was alluded to, rather, however, as poetical licences than as philosophical deductions. We are glad to perceive, that the Bishops and other non-professional visitors who were present considered the learned President's paper, as supporting the doctrine of the immortality of the soul and the fundamental tenets of our holy religion—thus tending to wipe off the stain of scepticism, so long attached to medical philosophy.

For our own parts, we are almost

---

* See Medical Gazette, Feb. 13th, 1830.

ashamed to say, that this first soirée of the College led to a train of reflexions much less celestial than the President's paper was calculated to inspire. Sir Henry Halford is possessed of an influence capable of effecting a work of great benefit to the profession and honour to himself. By his personal influence the Charter of the College might be enlarged, re-modelled and placed upon a basis corresponding with the present state of Europe—a basis that would extend and augment its power, by an accession of talent and learning from what may be called the democracy of the profession, but which would make it the first scientific institution in the world Has the constitution of England been weakened by an admixture of aristocracy and democracy? What would the House of Lords be, without that of the Commons? Why should not the Licentiates of the College have some tie or connexion with it, beyond the mere *permit* to carry on the practice of their calling? The eagle-eyed President cannot be unaware that the immense body of Licentiates labour under the same disabilities as the Catholics lately did—that they must consider themselves as degraded outcasts from the College—and can never, under such circumstances, cordially unite in supporting its splendour or respectability. The annual, biennial, or triennial election of dying talent, or of a Court favourite, is a sorry boon to merit, which sycophants may extol, but which the independent mind can never commend. The enlightened President knows this, as well as he knows that the sun rises in the east and sets in the west. But he is fettered, not so much by aristocratical prejudices, as by aristocratical associates. The fleeting moment is rapidly passing away, in which he might lay the foundation of an edifice that would associate the name of Halford, with that of Harvey and Hunter, to the latest records of posterity! That moment past, the golden opportunity will vanish; and future generations may peradventure, quote these lines, when the place where the dust of President and Prophet is interred, shall be totally forgotten.

